# A Case of Primary T-Cell Central Nervous System Lymphoma: MR Imaging and MR Spectroscopy Assessment

**DOI:** 10.1155/2013/916348

**Published:** 2013-05-23

**Authors:** G. Manenti, F. Di Giuliano, A. Bindi, V. Liberto, V. Funel, F. G. Garaci, R. Floris, G. Simonetti

**Affiliations:** Department of Diagnostic and Interventional Radiology, Molecular Imaging and Radiation Therapy, Policlinico Tor Vergata, Viale Oxford 81, 00133 Rome, Italy

## Abstract

Primary central nervous system lymphomas (PCNSLs) are mainly B-cells lymphomas. A risk factor for the development of PCNSL is immunodeficiency, which includes congenital disorders, iatrogenic immunosuppression, and HIV. The clinical course is rapidly fatal; these patients usually present signs of increased intracranial pressure, nausea, papilledema, vomiting, and neurological and neuropsychiatric symptoms. PCNSL may have a characteristic appearance on CT and MR imaging. DWI sequences and MR spectroscopy may help to differentiate CNS lymphomas from other brain lesions. In this paper, we report a case of a 23-year-old man with T-primary central nervous system lymphoma presenting with a mass in the right frontotemporal lobe. We describe clinical, CT, and MRI findings. Diagnosis was confirmed by stereotactic biopsy of the lesion.

## 1. Introduction

PCNSL causes approximately 3%-4% of all primary brain tumors. PCNSL is defined as lymphoma in the central nervous System (CNS) without primary tumor elsewhere. PCNSL is mainly diffuse large B-cell lymphoma; less common PCNSL histological types are Burkitt's lymphoma and T-cell lymphoma. 

Our case is about a 23-year old man with T-cell central nervous system lymphoma (T-PCNSL).

## 2. Clinical History

A 23-year-old man was referred to our emergency room because of nausea, vomiting with associated acute confusional state. Neurological examination revealed slow response, postural instability without rigidity or tremor in any of the four extremities, and normal sensation. 

No remarkable abnormality was observed at the physical examination. 

Laboratory tests were within normal limits, in exception for LDH 61,1 mU/mL (5–36 mU/mL normal range). 

Past medical history was negative for considerable pathologies. 

HIV status of the patient was positive. 

Computed tomography (CT) of the brain revealed a 6 × 5 cm mild hyperdense mass in the right frontotemporal region with associated perilesional oedema. Mild mass effect on the homolateral ventricle was observed with 1 cm midline shift. 

**Figure 1:**
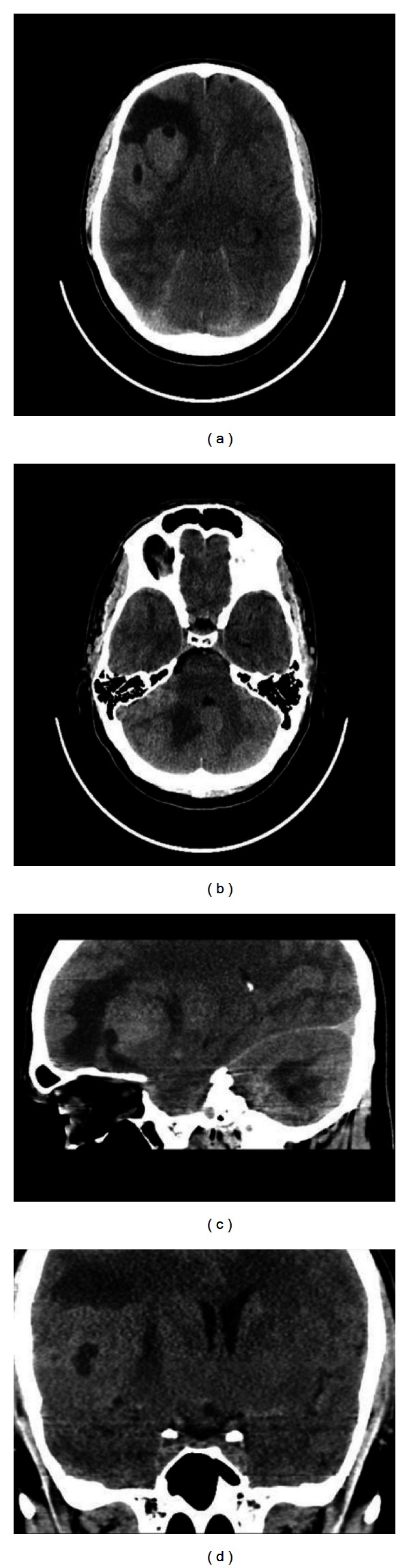
Axial CT acquisition (a)-(b) with sagittal (c) and coronal (d) multiplanar reformatted images showing a mild hyperdense complex lobulated mass in the right frontotemporal region associated with oedema and a similar smaller lesion in the right cerebellar lobe.

CT also revealed the presence of another similar lesion measuring 3,5 cm localized in the right cerebellar lobe (Figure 1). 

In the next, CT scan was integrated with brain MRI and single-voxel (1)H MR spectroscopy. 

**Figure 2:**
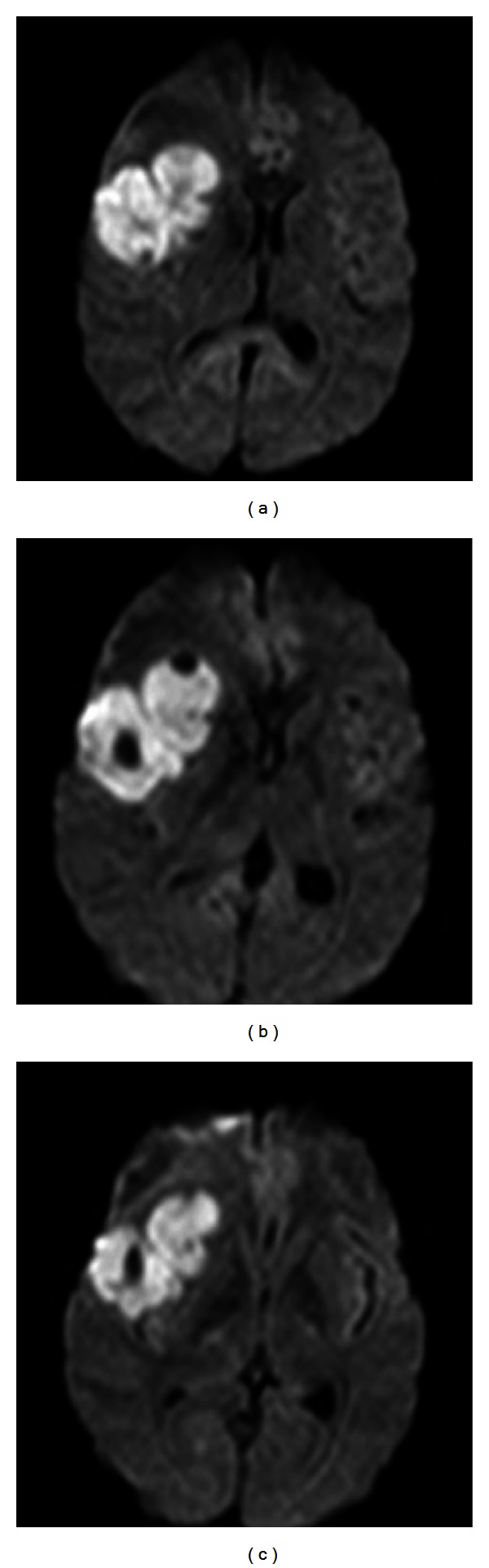
Isotropic diffusion weighted images (DWI) showing signal restriction associated with the fronto-temporal lesion.

Diffusion weighted imaging (DWI) showed hyperintense lesions at the right fronto-temporal area and at the right cerebellar lobe, respectively (Figure 2); apparent diffusion coefficient (ADC) map confirmed the presence of hypointense lesions. 

**Figure 3:**
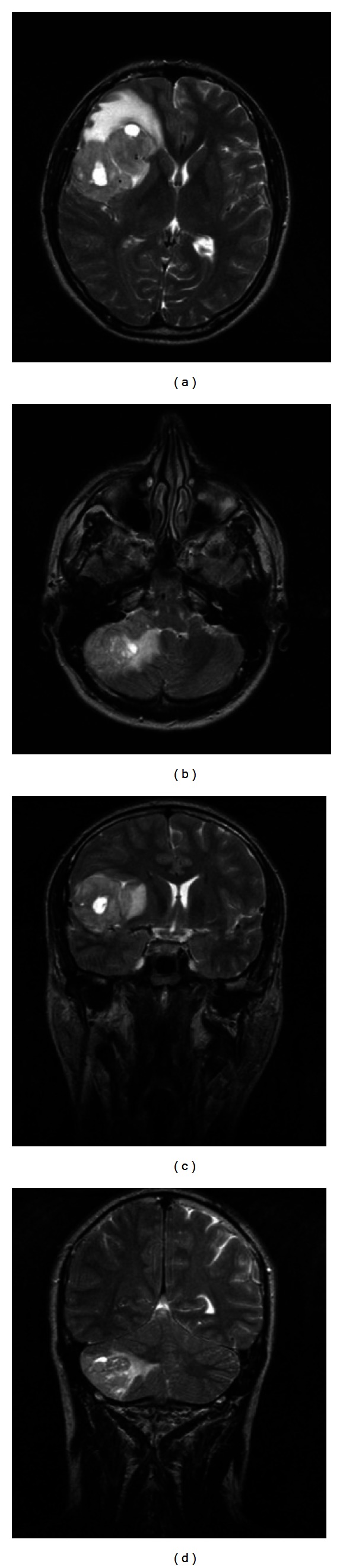
Axial and coronal T2-weighted images showing inhomogeneous hyperintensity of the cerebellar and fronto-temporal right lobe lesions associated with ovalar hyperintensities and perilesional oedema.

Masses appeared mildly hyperintense also in T2-weighted MR images with peri-lesional oedema (Figure 3). T1-weighted images showed the presence of multiple millimetrical cystic formations in the center of the huge sovra-tentorial lesion. 

**Figure 4:**
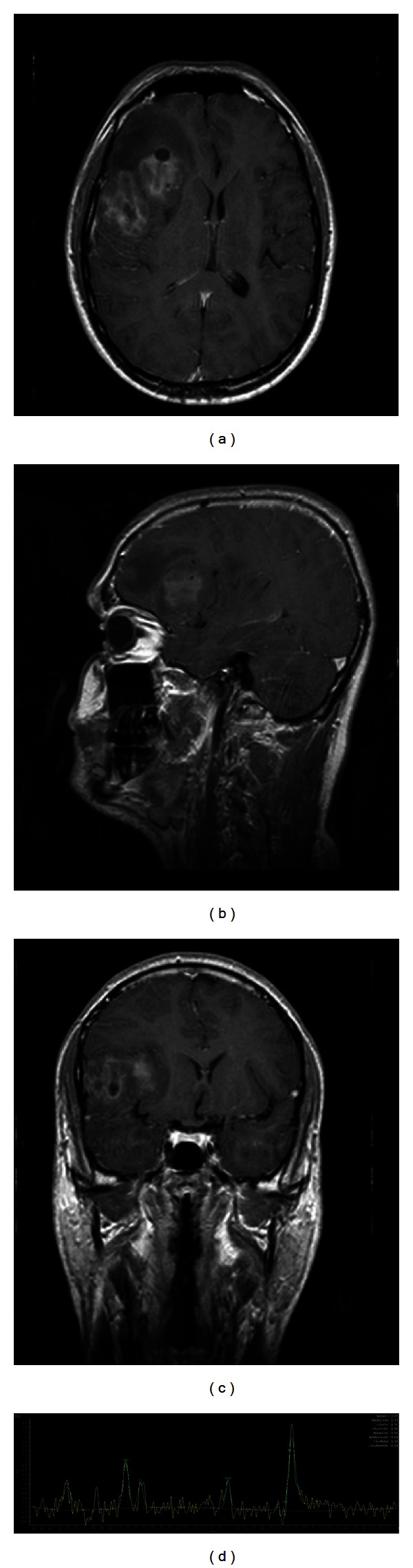
Gadolinium-DTPA enhanced T1-weighted multiplanar images (a), (b), and (c) reveal heterogenous enhancement throughout the fronto-temporal mass; single-voxel H MR spectroscopy (d) shows a lactate/lipid peak, increase of Choline/creatine ratio, and depression of N-acetylaspartate/creatine ratio in the fronto-temporal lesion.

Gadolinium-DTPA enhanced T1-weighted images revealed mild heterogenous enhancement throughout the masses (Figure 4). 

Single-voxel (1)H MR spectroscopy was then performed to evaluate metabolic information of the tumor. The (1)H MR spectra were obtained with long TE acquisition, a single-voxel point resolved spin-echo sequence for localization (TR/TE 2,000/144 ms) and a three-pulse chemical shift selective saturation sequence to provide water suppression. (1)H MRS was characterized by a lactate/lipid peak, increased Choline/creatine, and reduced N-acetylaspartate/creatine ratios in the lesion (Figure 4). 

Staging of the disease was performed using CT scan with no distant metastases shown at different levels. 

Cerebrospinal fluid examination revealed white cell count 1/*μ*L, glucose 80 mg/dL (blood glucose 110 mg/dL), proteins 45.1 mg/dL (reference: 8–32 mg/dL), lactate 15.3 mg/dL (reference: 10.8–18.9 mg/dL), and IgG index 0,95 (reference: <0,5). 

Stereotactic biopsy of the right frontal lobe was performed showing medium-sized lymphoid cells with a perivascular pattern. Immunohistochemical showed positive staining of CD3, CD8, and CD56 indicating that tumor cells were T-cells lymphoma in origin. 

EBV (EBER) status of the lesion was not included. 

Flow cytometry study of the peripheral blood and bone marrow supported the notion of no systemic involvement. 

The diagnosis was T-primary central nervous system lymphoma (T-PCNSL). 

In light of the lack of any potentially effective radiation option, the patient underwent chemotherapy with methotrexate at high dose of 3500 mg/mq and intrathecal administration. 

The radiation oncologists were consulted and proposed radiotherapy consolidation with a total of 45 Gy divided in 1.8 Gy/die on the entire brain, extended to C2. 

## 3. Discussion

Primary central nervous system lymphomas (PCNSLs) are rare non-Hodgkin tumors without any evidence of systemic lymphoma. PCNSL represents 3%-4% of primary brain tumors and 1%-2% of all lymphomas. In most cases are diffuse B-cell lymphoma, T cell is very rare constituting of 1,7% of all PCNSLs [1, 2]. 

The overall incidence rate of PCNSL is 4 cases per million persons per year. The peak incidence is between 60 and 70 years old for immunocompetent patients. The male: female ratio is 1.5 : 1 [3, 4].

Young age of the patient should be noted, because it is not typical for lymphoma. Significant increment of incidence rate over time is associated with increased incidence of AIDS and advanced age. 

Prominent risk factor for the development of PCNSL is immunodeficiency, which includes congenital disorders, iatrogenic immunosuppression and HIV [5, 6]. 

PCNSL usually presents, solitary or multiple lesions mainly located at supratentorial level, usually in the periventricular regions, infiltrating the corpus callosum and the basal ganglia. 

Multiple lesions are reported in 38%–55% of non-AIDS PCNSLs. Multifocal intraparenchymal lesions without a dural involvement are very uncommon. Frontal lobe is affected in 20%–43% of PNCLs, brain stem, or cerebellum in 13%–20%. Other localitations are leptomeninges, spinal cord, and eyes [7]. 

Our patient had multifocal lesions located in right frontotemporal lobe and in homolateral cerebellar lobe with dural involvement.

Patients with PCNSL usually present with neurological and neuropsychiatric symptoms. Neurological examination reports pyramidal signs or sensory abnormalities associated with extrapyramidal symptoms, including rigidity, bradykinesia, and masked face. Headache is frequent (56%) as like as other signs of increased intracranial pressure, such as nausea (35%), vomiting (11%), and papilloedema (32%). 

Changes of personality, irritability, restlessness, and inappropriate behavior are common, since the tumor shows a tendency to be located at the frontal lobes [8]. 

In the literature, multifocal tumors present symptoms at an earlier stage because multiple lesions are likely to have a larger effect than single lesions [9]. 

CT has been the primary method for the evaluation of the PCNSL. Tumor results in hyperdensity, but it may also appear isodense (Figure 1). However, CT is not a gold standard technique for diagnosis because a negative examination does not exclude CNS lymphoma and 13%–38% false-negative rate is reported [7]. 

Magnetic resonance imaging offers several potential advantages in the evaluation of these lesions by using DWI sequences and MR spectroscopy for differential diagnosis. T-cell PCNSL usually appears with a subcortical distribution, peripheral nervous system involvement, and leptomeningeal spread. 

On unenhanced T1-weighted imaging, lesions are typically hypo- or isointense and on T2-weighted MR imaging iso- to hyperintense to gray matter (Figure 3). Most lesions show moderate-to-marked contrast enhancement [7]. Only in some rare cases of PCNSL MR imaging shows isolated white matter hyperintensity on T2-weighted sequences with no contrast enhancement on T1-weghted MR imaging. 

Typical features of PCNSL are “notch sign,” an abnormally deep depression at the tumor margin and “open ring” enhancement. There could be mild or marked perilesional oedema [2]. Hemorrhage or internal calcifications within the tumor are a quite rare finding [7]. 

Because CNS lymphomas are highly cellular tumors, water diffusion is often restricted, making them appear hyperintense on DWI and hypointense on ADC maps (Figure 2). Differential diagnosis of lesions with these features is with ischemic stroke, central necrosis of brain abscess, high-grade gliomas, or some metastases. Doskaliyev et al. suggested the possibility to differentiate lymphoma from glioblastoma by means of ADC values [10].

ADC is inversely associated with tumor cellularity with lymphoma lower than glioblastoma [11].

In addition to morphological MRI, (1)H-MRS provides noninvasively a wide spectrum of biochemical information with the lesion, which can be used for differential diagnosis of expansive lesions, estimation of the tumor type, and therapeutic response monitoring [12–14].

In PCNSL, proton MR spectroscopy was characterized by predominance of lipid peaks combined with high Cho/Cr ratios and reduced N-acetylaspartate (Figure 4) [1, 7, 15]. 

These findings are reported on glioblastoma multiforme and some metastates with definitive diagnosis reliant on histopathology. 

Histology showed an infiltrate comprised of small, intermediate, or large-sized lymphocytes with surrounding plasma cells in a perivascular configuration with associated background reactive atrocities and Rosenthal fibers. 

Lymphocytes can show nuclear membranes irregularities, moderately dispersed chromatin, inconspicuous nucleoli, and perinuclear halo. Necrosis can be noted. 

On immunohistochemistry the lymphocites show positive staining for CD3, CD8, and CD56 but negative staining with CD2, CD5, CD7, CD4, CD20, and CD30. 

Unfortunately T-cell gene rearrangement study was not included. 

PCNSL has a 5-year survival rate between 4% and 40%. The clinical course is rapidly fatal if treatment is not promptly started. Surgery has not improved the survival, with an average of 3.5–5 months and a deterioration in the quality of life [8]. 

The standard treatment for PCNSL has not been defined yet for the lack of adequate randomized studies. Retrospective series have shown a very significant survival advantage for the combination chemoradiotherapy. First-line chemotherapy consists in high-dose methotrexate followed by radiotherapy. This strategy allows a 5-year survival of 25%–40% versus 3%–24% with the radioboost alone [16]. 

## 4. Conclusion

T-PCNSLs are extremely rare brain tumors that affect the elderly and immunocompromised patients 

DWI MRI sequences have some pathognomonic aspects which may help differential diagnosis between brain lymphoma and other glial tumors. Contrast-enhanced MRI can actually be considered the gold standard imaging technique. 

In addition to morphologic MRI, (1)H-MRS provides non-invasively a wide spectrum of biochemical information of the lesion. 

CT-guided biopsy, with immunohistochemical sample studies, should be thoroughly performed.
